# Research agenda for using artificial intelligence in health governance: interpretive scoping review and framework

**DOI:** 10.1186/s13040-023-00346-w

**Published:** 2023-10-31

**Authors:** Maryam Ramezani, Amirhossein Takian, Ahad Bakhtiari, Hamid R. Rabiee, Sadegh Ghazanfari, Saharnaz Sazgarnejad

**Affiliations:** 1 Department of Health Management, Policy and Economics, School of Public Health, Tehran University of Medical Sciences, Tehran, Iran; 2 Health Equity Research Centre (HERC), Tehran University of Medical Sciences, Tehran, Iran; 3 Department of Global Health and Public Policy, School of Public Health, Tehran University of Medical Sciences, Tehran, Iran; 4 Department of Computer Engineering, Sharif University of Technology, Tehran, Iran; 5 School of Public Health, Tehran University of Medical Sciences, Tehran, Iran; 6 School of Medicine, Tehran University of Medical Sciences, Tehran, Iran

**Keywords:** Artificial intelligence, Health system, Governance, Stewardship, Framework

## Abstract

**Background:**

The governance of health systems is complex in nature due to several intertwined and multi-dimensional factors contributing to it. Recent challenges of health systems reflect the need for innovative approaches that can minimize adverse consequences of policies. Hence, there is compelling evidence of a distinct outlook on the health ecosystem using artificial intelligence (AI). Therefore, this study aimed to investigate the roles of AI and its applications in health system governance through an interpretive scoping review of current evidence.

**Method:**

This study intended to offer a research agenda and framework for the applications of AI in health systems governance. To include shreds of evidence with a greater focus on the application of AI in health governance from different perspectives, we searched the published literature from 2000 to 2023 through PubMed, Scopus, and Web of Science Databases.

**Results:**

Our findings showed that integrating AI capabilities into health systems governance has the potential to influence three cardinal dimensions of health. These include social determinants of health, elements of governance, and health system tasks and goals. AI paves the way for strengthening the health system's governance through various aspects, i.e., intelligence innovations, flexible boundaries, multidimensional analysis, new insights, and cognition modifications to the health ecosystem area.

**Conclusion:**

AI is expected to be seen as a tool with new applications and capabilities, with the potential to change each component of governance in the health ecosystem, which can eventually help achieve health-related goals.

## Background

In general, healthcare systems can affect the population's health status by a maximum of 20% [1]. In other words, besides access to healthcare services, the existence of a variety of external factors including socioeconomic, political, and cultural issues, play a key role in the mortality and morbidity of populations. In this line, particular attention has been paid to the failure of the typical responses of healthcare systems [2]. Meanwhile, Stewardship, as a core building block of the health system [3, 4], involves a complex combination of activities that run concurrently at multiple different levels. These activities can vary from strategic (legislation) to mechanical (financial or clinical). They might involve a variety of institutions and health system levels in any process [5, 6]. The World Health Report (2000) described stewardship as ‘the careful and responsible management of the population's well-being’. Undoubtedly, the governance of health systems should benefit from the advantages of technological advancements such as AI [2, 7]. AI uses computer simulations to mimic an intelligent behavior with little or no human involvement [8] to face current and future challenges [2]. AI is defined as ‘the ability of a system to act appropriately in an uncertain environment, which increases the probability of success and supports the system's ultimate goals’ [9]. Big data analytics as one of the AI advancements, utilizes a cluster of computers to process the enormous volume of unstructured data in a concurrent and distributed computing environment [10]. On the other hand, AI is about creating an upheaval in the world, which can change how we live [11]. Currently, AI has become a helpful tool for countries and governments to make changes and enhance public governance as a paradigm shift from traditional public governance. AI can improve cooperation between government and social organizations to supply public goods, which can lead to optimizing resource allocation and improving innovative approaches [12]. Further, AI can be a beneficial tool to increase the effectiveness and efficiency of governance in environmental management [13, 14]. AI-assisted governance tools enable the public to be involved in policymaking and be ensured about transparency and accordingly enable smart governance [14]. AI methods are employed to explore the governing factors and their thresholds, for instance through real-time monitoring to provide early warning that eventually can be beneficial to prevent disasters and their consequences [15]. Furthermore, AI makes it possible to identify factors that contribute to the complexity of health problems at the national and international levels [16]. AI impact the way that decisions are made in health systems, and it even will be having more impact in the coming future [17], Therefore, this study aimed to investigate the roles of AI and its applications in health system governance through an interpretive scoping review of current evidence.

## Method

This is a scoping review guided by Arksey and O'Malley's framework, including 1-identify the research question 2-identify relevant studies 3-study selection 4-data charting, 5-summarizing and reporting the results, and 6-expert consultation has been performed [18].

Following the first step, the following research questions were formulated: 1- Which applications of AI in health system governance are supported by the literature?; 2- What future applications and capabilities can be employed regarding health system governance?. Then, using appropriate keywords, we identified relevant studies. Two authors (MR & AB) conducted a comprehensive literature search through three databases: PubMed, Web of Sciences, and Scopus independently, and screened the titles and abstracts of articles in terms of relevancy. We reviewed the full texts of relevant articles, chartered the extracted data and collated, summarized, and reported the collected data. Finally, the synthesized results were presented, discussed and approved by consensus of the corresponding author and the entire research team.

### Search strategy

#### Databases and keywords

We searched PubMed, Scopus, and Web of Sciences databases were searched using the appropriate combination of keywords related to governance and AI. Table 1 presents the search queries used for target databases.

**Table 1:** Databases and keywords

Databases	Query	Initial results
PubMed	("governance"[Title] OR "government"[Title] OR "stewardship"[Title] OR "administration"[Title] OR "management"[Title]) AND ("data mining"[Title/Abstract] OR "big data"[Title/Abstract] OR "artificial intelligence"[Title/Abstract] OR "deep learning"[Title/Abstract] OR "machine learning"[Title/Abstract])	1169
Web of sciences	((((AB = ("machine learning")) OR AB = ("big data")) OR AB = ("data mining")) OR AB = ("deep learning")) OR AB = ("artificial intelligence") ((((TI = (governance)) OR TI = (administration)) OR TI = (stewardship)) OR TI = (government)) OR TI = (management) AB = (Health)	560
Scopus	( ( TITLE-ABS-KEY ( "big data") OR TITLE-ABS-KEY ( "data mining") OR TITLE-ABS-KEY ( "deep learning") OR TITLE-ABS-KEY ( "artificial intelligence") OR TITLE-ABS-KEY ( "machine learning"))) AND ( TITLE-ABS-KEY ( health)) AND ( ( TITLE ( governance) OR TITLE ( administration) OR TITLE ( stewardship) OR TITLE ( government) OR TITLE ( management))) AND ( LIMIT-TO ( PUBYEAR, 2023) OR LIMIT-TO ( PUBYEAR, 2022) OR LIMIT-TO ( PUBYEAR, 2021) OR LIMIT-TO ( PUBYEAR, 2020) OR LIMIT-TO ( PUBYEAR, 2019))	1433

### Inclusion criteria

We included studies published in English on the governance of health systems (inclusively or exclusively) from 2000 to 2023. Articles without full-text were excluded.

### Charting data

**Fig. 1:**
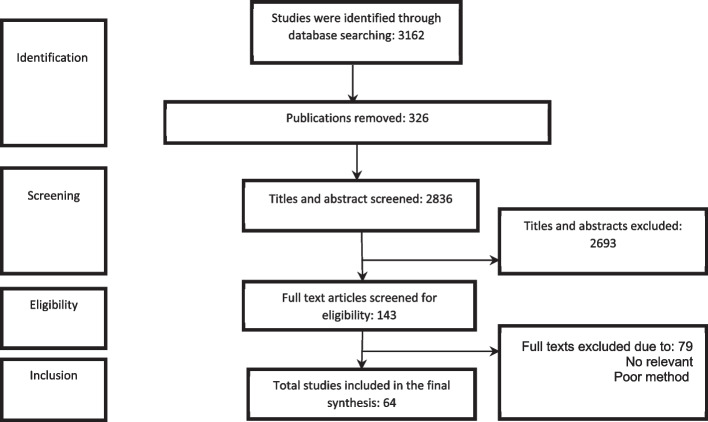
Flow chart of the search strategy

We synthesized the data by placing similar codes into categories of AI applications in health system governance and proposed a conceptual framework. To develop the framework, two authors (MR and AB) independently categorized AI applications from the identified articles and created descriptions by synthesizing extracted information (Fig. 1). All authors reviewed and discussed the framework until we reached final consensus.

## Results

Our initial search identified 3162 references, 326 of which were duplicated. We screened a total of 2836 articles with title reviews, which resulted the exclusion of 2694 articles. 143 studies met the inclusion criteria for full-text evaluation, and 79 studies were excluded due to the lack of relevancy or poor methodological quality.

**Fig. 2:**
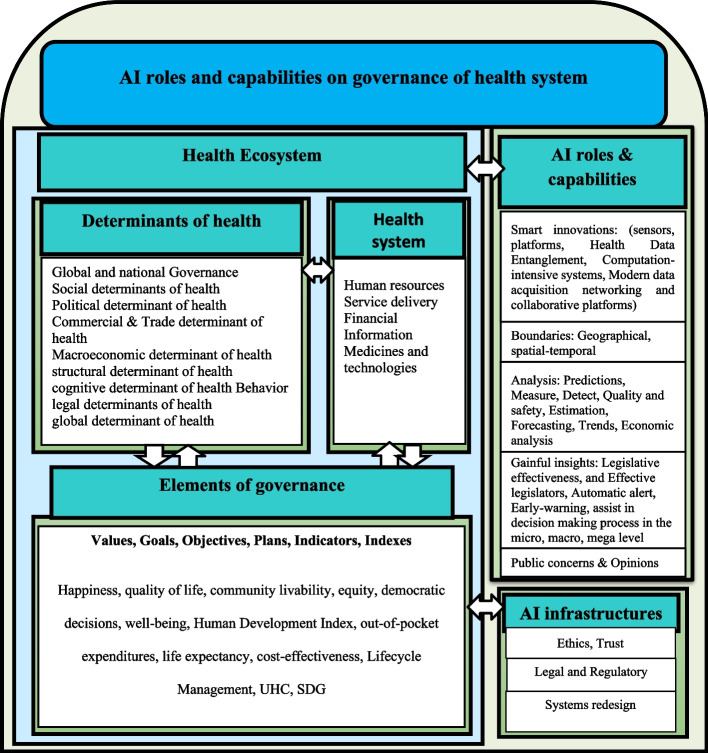
AI applications and capabilities for health system governance (contemporary studies and future agenda)

The results are presented in two parts: a summary of the extracted information (Appendix 1); a proposed framework to explain its dimensions (Fig. 2).

### A Framework for future studies: existing knowledge and future directions

As shown in Fig. 2, the proposed framework has two main components: a sub-component for AI and three sub-components for the health system. The conceptual framework shows that AI can affect health ecosystems with various applications and capabilities.

### AI applications and capabilities

In the realm of public health decision support systems, toolkits and platforms such as CrowdHEALTH have been introduced [19, 20]. Besides, data mining and machine learning-based data analysis can yield in-depth details and in-time information generation, which can lead to effective management of specific areas [21]. The worldwide emergence of Intelligent systems such as smartphones and watches [9] and other modern tools [22, 23], can provide new values in data collection. Given the rapid and impressive progress of these technologies’ improvements in the collection, extraction, processing, analysis, and prediction of medical information, more revolutions are expected in the future [24]. These intelligent systems must be able to continuously collect real and near-real data, create knowledge, and legislate a set of activities [25]. Therefore, AI can be used to detect significant events, public concerns, and government measures and analyze issues related to time and space [26]. One study proposed a system that patients with chronic diseases can continuously receive active recommendations related to their healthcare through rule management. In addition, the incidence of secondary diseases can be prevented, and health management could be performed by reference to patient-specific lifestyle guidelines [27].

Detection and tracking trends in social media is a valuable action [28]. Microblog data including semantic and sentiment vocabulary of words can be analyzed by AI, and novel outlier knowledge can be obtained that serves as a think tank to prevent and control sudden incidents [29]. Besides, new related applications can be used to analyze big data such as Google or rolls, or regulations [25], and legal data [30] which can ultimately reduce political roles and improve new types of interventions and achieve timely actions during health emergencies' occurrence such as pandemics [31], perhaps through sending automatic alerts [25].

AI can also address factors that cause complex health challenges at the macro level, including governance and socioeconomic factors, by recognizing health-related problems [32], building a smart world both for the private and public sectors [33], and providing public opinion mining [16, 34]. Making timely and preventive decisions will be possible by enhancing effective forecasting, supporting early warning systems, impact evaluation of determinants of health, and interventions cost-effectiveness analysis (CEA) [25, 31, 35].

Regarding environmental issues, one study presented a novel geospatial platform for green space area management, using geographic information systems (GIS) and state-of-the-art AI technologies [36]. In another study, to deal with public health events, a framework of outlier knowledge management was developed based on three main aspects including situation, dimension, and object [29]. Further, AI capabilities such as spatial and temporal-based analyses [10] can be used to achieve worldwide pillar goals [22], and indicators at macro and micro levels [16], to support decision-making at both national and global scales [37]. Structural topic modeling (STM) can be applied to a text to reveal topic network and topic trends to perform analyses that are based on frequency, proportion, and importance over time and space (conceptual, temporal, and geographical trends [38]. Participation of different actors based on geographical units that affect health inputs or outputs, such as achieving goals, disease burden, and health costs, provide a new situation for the system [16]. This approach can reveal the scientific evolution of research subjects and their trends and therefore, provides an interactive user interface for further analyses [39].

### AI infrastructures

While recent technologies, particularly AI, constantly modify human interactions, a fundamental question is: ‘how to ensure digital resilience and collective well-being, while safeguarding liberal democracy and individual rights’ [40]. To ensure the provision of beneficial answers regarding the mentioned concern, new capacity building in institutions are needed to implement such efforts at national and international levels [41]. Appropriate technologies, facilities, particular expertise both for users and big data experts [42], shared guidelines for databases [43], norms, public relations, and education would remain important factors for the successful adoption of modern technologies. The legitimacy of such technologies should be debated as well [25]. The relationship between actors will change in these new systems; which might ultimately reduce physicians’ power as the main rulers of the health system [44].

According to IBM's estimation, in 2017, 90% of the available global data on AI were generated in previous years [43]. Collecting large amounts of data in various forms (e.g., photos, audio, video, and files) [42] from areas that affect or are affected by health, would require capacity-building [42]. These areas could be related to education, transportation, agriculture, food safety, and security, as well as the incidence of emergencies [45], genomic [43], antimicrobial, and demographics [45] and, eventually, be linked to create an aggregate system [32, 46]. Meanwhile, regulation of this burgeoning field must explicitly address and call out health disparities [47]. Moreover, the challenge is a greater risk for legal regulations to keep up with the accelerated global changes resulting from Big Data, and the loss of information privacy created by digital transformation [48]. One study identified three main ‘‘trust facilitators’’ of AI, including (1) technical, (2) ethical, and (3) institutional [49, 50]. The challenge for Health information management (HIM) professionals will be to develop practice standards for the management of healthcare data and information in an AI-enabled world [17].

Despite several applications, various studies are needed on data security and privacy to prevent negative consequences in the future [42, 51] and construct responsible AI [32]. Although technological advancement will improve the health status, new requirements on transparency and accountability, as well as protection of fundamental human rights, will be required. These concerns need precise planning, which needs to be protected by national legal systems and regulations [44], which might pave the way for opening the door to the idea that AI should have political power [52].

### Stewardship & Governance

#### Values, goals, objectives, plans, indicators, and indexes

Indices and indicators such as happiness [53], quality of life [54], community livability [38], equity [22], and democratic decisions through citizen participation [55] have been discussed in previous AI studies. AI can be used to improve goals, objectives, or indicators at both national [31] and international plans. Pre-screening could alleviate an administrator’s workload by screening crowdsourcing data and automatically removing irrelevant submissions [36]. Benchmarking and collaboration, not only at local and national levels, [23] but also at the level of international governance, provide capacities for policy learning. More intelligent decisions and citizen participation, can lead to improved transparency, responsibility, and democracy [55]. Social responsibility [22] and multivariate co-governance [30, 51] create new capacities, which need new structures for institutions to manage macro-level problems [22].

Stability and harmony of society, economy, and environment are followed by AI towards achieving sustainable development goals (SDGs), [30, 32, 56], as well as the international and national priorities [57], e.g., analyzing safety, quality of agricultural products, [30] pollution and their impacts on health and economic losses from different aspects such as losses due to lost work, and medical expenses due to illness [30, 58]. A study proved geospatial system as a viable tool for assisting governmental agencies to devise appropriate plans toward SDGs [36]. Big data is a cue to improve the effective implementation of government policies, particularly in the tax, healthcare, education, and culture sectors of the government’s jurisdiction. It can also shed light on hidden disparities embedded in societies and enable more agile, efficient, and evidence-based decision-making [59].

AI can distinguish and predict various features that contribute to well-being [53, 54]. It is worth mentioning that, creating social and environmental values can help manage stakeholders' conflicts of interest [42]. Changes in cognitive aspects of determinants of health, even in decentralized institutions, might change the legitimacy of related organizations [23, 31]. Opinions of individuals and officials [34] can also be collected from different forms of data. In the future, AI tools will help rationalization of the values and beliefs of public authorities and health actors. Big data strategies can transform government and public services to become more citizen-centric, responsive, accountable, and transparent [59].

Information and communication technology-based applications such as e-government [55, 60], smart city, and smart health [31, 59] will find unique applications for the governance of the health ecosystem with AI [61]. Data analytics uses historical data to provide intelligent solutions to make better decisions and strategies for the betterment of society [60]. Platforms such as MIDAS can be designed to analyze data like city and government-generated datasets (e.g., at health, social care, mainly individual personal levels), open government data (air and water quality), national statics (education, unemployment), or urban planning to address the priority of health policies. Using these platforms can help better understand diseases and their impact. Moreover, it will facilitate monitoring different aspects of diseases across groups, resiliency against global crises, and preparedness for future emergencies [62]. Eventually, AI will foster efficient and agile policies to preserve the environment and efficient utilization in nurturing sustainable development and promoting welfare [42], which will improve democracy and enhance trust in governance systems [63]. Some scholars also argue that future democratic innovation will be markedly different, which calls for a better understanding of how institutions and governments can integrate digital technologies and data science approaches into public discourse, to let the voices of the people be heard regardless of their socioeconomic status, party affiliation, or party(ies) in power [40].

### Determinants of health

#### Business

AI can provide efficient strategies for businesses that might have conflicts of interest with environmental sustainability [42]. It can then be extended to other related subjects to avoid negative consequences.

#### Technical

AI can be used to identify a systematic association among various factors. In this well-defined ecosystem, it is easier to deal with diversity and complex situations by understanding rules and regulations, designing exclusion, exceptions, and thresholds, and introducing features in a cost-effective and timely manner for stakeholders to detect and respond to non-compliance [25]. For instance, GIS software is needed to provide predictive analysis, which can lead to effective planning and optimization of management operations. Such analyses allow policymakers to devise policies that are cost-efficient and optimal in terms of financial and human resources [21].

#### Global

AI can be used for hygiene, infection control, and vaccination at local, national, and international levels [43]. AI-based analysis and Health Data Entanglement (HDE) can improve the effectiveness of health governance, which can lead to high-quality healthcare services [9]. These applications cause successful health strategies such as vaccination, which has national and international health effects [64] with greater consideration of disparities [65]. A retrospective analysis of mobility patterns showed that social distancing behavior occurred before the start of government stay-at-home orders [66].

#### Social

AI creates new types of social [5] and local governance [33]. It facilitates the remote provision of services, education, and service delivery, which is effective in normal circumstances as well as during pandemics (e.g., better implementation of social distancing or lockdown) [33]. As the behavioral perspectives between socio-demographic and personal attributes of the population are different, AI analysis can lead to more effective citizen management [67]. Moreover monitoring the progress of interventions allows the efficient availability of the measure in real-time [68].

Early identification of sub-populations simultaneously could help improve people’s quality of life and reduce healthcare costs. In another study, a population health management tool for the early identification of high-risk patients based on state-of-the-art intelligent algorithms has been described [69].

#### Environment

AI can improve understanding of climate change, help combat climate crisis [70], estimate air quality, while allowing the combination of multidimensional attributes [56, 71]. Using multiple data can improve environmental governance and management of emergencies [37]. PRAISE-HK is an AI system based on real-time data on emissions [72] that can be extended to assess the association between social-economic-environmental systems [73].

AI has a great potential to analyze primary business data, media, and regulations [51] to determine the areas in need for more investment [22], which might help authorities for effective planning [21]. One study developed an AI system to improve the management of sensors, short and long-term maintenance plans, and asset and investment management plans in the area of water, wastewater, and reuse plants using the IViewOps (Intelligent View of Operations) model [74].

#### Economic

AI allows economic loss estimation based on exposure to pollution, and its impacts on health status. Different health-related outcomes including mortality, individuals' daily functions, emergency cases, children with lower respiratory tract infection/asthma, outpatient rate of respiratory disease, asthma cases, and chronic bronchitis can be considered [58]. In another study, researchers explored the influencing factors of air pollution on government health expenditure and spatial governance [75]. Through such analyses, investment and budget spending in projects related to high-priority determinants of health can be distinguished. Hence, donor governments and civil society can estimate the financial assets allocated to each part [22]. AI could provide greater opportunities to analyze neglected social costs, cost per QALY, and economic sustainability and cost-effectiveness evaluations. Another study discussed HDE to analyze subjects related to gross domestic product (GDP) growth, sustainably, and industries [9].

#### Political

The multifaceted and complex decisions of political systems, especially in critical situations, can be influenced by AI applications to save costs, seek synergies, and find opportunities [76]. In this regard, based on a former investigation, the benefits of better political decision-making through AI are considerable. AI is already being employed in the public sector to improve political analyses and decision-making processes. If we agree that politics should have a good policy as a goal, and if we do not consider our current democratic order as a given and inviolable good, we may begin welcome AI to have political power [52]. AI can provide critical information, assist the political decision-making process, and contribute to collaboration among stakeholders to avoid tradeoffs between them and pooling the resources more efficiently [76], hence it contributes to the health ecosystem governance with a new viewpoint.

#### Legal

Given its significant capacity and potential, AI can facilitate the implementation of laws and regulations in different dimensions of health determinants. For instance, one study explored the mechanisms of law enforcement of agricultural quality or safety to improve traceability and amend laws for building various legislative systems, formulating the standards of supervision, establishing networked supervision systems, and enhancing public awareness to support society's leadership [30].

### Health system

#### Goals, indicators, and plans

AI is applicable to solve complex environmental and social life problems by integrating different inputs and Indicators, e.g., the government effectiveness index, economic policy, the share of the health sector from GDP, the rule of law, health expenditure per capita, control of corruption index, Human Development Index, and out-of-pocket expenditures [16, 23, 38, 77].

Wearable technologies are useful tools in the field of healthcare, particularly for people with chronic diseases, like diabetes. They can help manage diabetes and reduce the incidence of related complications. Furthermore, the utilization of these devices has improved illness management and patients' quality of life. It can be argued that emerging digital technologies, big data-based analytics, and the vast application of AI to communicable and non-communicable diseases will revolutionize the prevention, treatment, and management of the diseases and their complications [78].

Hierarchical matrices can be used for the selection of appropriate interventions for individuals, which is useful to identify high costs and reduce costs for the member and insurer [79]. Gender-related aspects, as well as differences in exposure to adverse social and environmental circumstances, contribute to better prevention and development of gender-transformative care [80]. Such novel information and analyses have the potential to improve management [22]. With the support of big data, critical indicators could be tracked from the perspective of laws [30] and regulations. interventions can be designed with more agility, transparency, and responsibility of a domain [25] and as a result the trust and confidence of people [34] will improve. Minimizing waste and efficient use of health-related resources can lead to transparency and accountability [16]. In one article, researchers revealed the role of data-intensive sourcing in the rising accountability around value-based care, through practices of population health management [81]. In other words, AI can play a vital role in terms of accountability, regulatory quality, and government effectiveness in health systems' governance [64].

#### Functions

AI can help health system strengthening by facilitating the improvement of all World Health Organization (WHO)’s six building blocks, including service delivery; health workforce; information system; medical products, vaccines, and technologies; financing; and leadership and governance (stewardship).

#### Service delivery

AI applications will change features of the healthcare system and other socioeconomic and legal determinants. It can explain the effects of variations in governance practices such as tasks, processes, and structures, on a set of performance variables including hospital outcomes and the effectiveness of governing bodies [82]. Some countries have already begun to redesign their service delivery and are developing AI for the direct engagement of citizens in service delivery [44]. Advancements in AI pave the way for enhancing the self-management of diseases, personalized experiences, empowering patients, engagement of patients in the decision-making process, and their well-being. Besides, new arrangements can facilitate coordination between private and public actors, and enable integrated efforts to meet health needs in delivering public goods and services [44]. In the healthcare system, data analytics can be used for infrastructure development [60] which results in better resource management and health surveillance systems [83].

The traditional optimal control methods of treatment strategies are commonly applied to deterministic systems, instead of dynamic systems with uncertain errors. To avoid this, one study proposed a new evidence-based optimal control (EBOC) [84]. In the industry, Product Lifecycle Management (PLM) is the process of managing the entire lifecycle of a product from inception, through engineering design and manufacturing, to service and disposal of manufactured products. This approach can be applied in health processes with the most specific treatment for each patient according to their needs [85]. Furthermore, using big data for the management of healthcare organizations contributes to improving the quality of service, crisis management, and data management areas of decision-making processes [86].

#### Financing

Identification of the sources of costs is crucial for sustainable healthcare systems. AI can be used to identify the influential factors on the attainment of Universal Health Coverage (UHC) and SDGs, as well as the enabling policies to address them effectively and efficiently. In one study, researchers predicted universal healthcare attainment through health financial management in line with sustainable development in three BRICS economic blocks [87]. In another study, an automatic deep learning-based Auto Triage Management Framework has been developed for accurate modeling of patients’ disease progression risk as well as health economic evaluation [88]. To select members for intervention, another study used AI to optimize the risk score threshold at which members are assigned an intervention. Such techniques help select the optimal members for intervention programs, reduce overall costs, and improve outcomes [79].

Previous investigations have shown the roles of big data analytics in identifying unprecedented discoveries through the COVID-19 pandemic from different perspectives, such as biomedical and economic viewpoints [89]. clinical outcomes and costs before and after implementing plans [90], CEA [35], economic value, and social impact of regulation systems in operational functions of health management [91] can be also analyzed by AI. To increase the healthcare budget in an uncertain situation and tackle healthcare spending growth, HDE is the appropriate solution [9]. Another study used a large amount of data to analyze factors influencing the health system, including two main demographic and economic categories [45].

Another example of these changes is the role of AI in combating antibiotic resistance in line with reducing economic costs [43, 92]. AI can predict behaviors, for instance, the impact of interventions and policies such as financial incentives (i.e., pay for performance) on indicators, e.g., quality-of-service delivery [93]. External factor evaluation on economic losses [58] can avoid haze governance and help preventive plans [58]. Besides, AI can facilitate effective advocacy strategies for better healthcare financing.

#### Information system

Recently, medical real-world data (RWD) accumulated in medical information systems have been considered for both primary and secondary use of medical information. AI has been widely utilized to solve problems and challenges in standardizing, collecting, cleaning, and analyzing such data. A new era in medical care and clinical research is emerging due to these new technologies [24]. The use of resources such as business intelligence, data mining, analytics, and AI is important to recognize patterns or accumulation of knowledge about the demographic profile, medical needs, and behavioral characteristics of patients [91]. HIM practices that are impacted by AI technologies include: 1) Automated medical coding and capturing AI-based information; 2) Healthcare data management and data governance; 3) Patient privacy and confidentiality; and 4) HIM workforce training and education [17]. AI analysis allows the management of complex economic processes, solving problems, advancing medium and long-term planning, and carrying effective horizontal and vertical interaction, which help achieve the ultimate goal of the health system: providing quality health services for all in need [94].

Distinguishing interrelation among different factors [77], interdisciplinary data integration [32], and providing novel information or analysis such as spatial information [23] are other capabilities of AI. By administering AI, researchers could address health problems appropriately and decrease the burden in an emergency such as a pandemic in an integrated manner, while developing information systems to help other related issues [46]. It is concluded that the analysis of georeferenced information, linked to health information obtained through the data mining technique, can be an excellent tool for the health management of a health plan operator, which can contribute to the decision-making process in health [95].

#### Human Resources

AI technologies are not intended to substitute healthcare workers, but individuals who can adapt to new workflows and processes may replace those who cannot [17]. International standards in technology, human resources management, and aging societies can provide appropriate solutions to improve aging workforce problems [96]. AI helps improve the traditional and single algorithm of the existing human resource systems, as well as the performance of the human resource management recommendation system [97, 98]. AI will continue to impact the way decisions are made in healthcare [99]. The challenge for HIM professionals is to develop practice standards for the management of healthcare data and information in an AI-enabled world [17].

#### Medicines and Technologies

AI can foster individual-level interventions such as the rational use of medicines, through information exchange with the support decision-making databases. Decision algorithms that are increasingly deployed in precision therapeutics and evidence-based medicine can save costs in standard care [35]. One study analyzed the trends of AI systems in the management of medical technological processes and health-related quality of life (HRQoL) [100]. The development of a model for more efficient urgent lab specimen transport [101], and analyzing medical costs associated with inappropriate use of medicines can help healthcare providers evaluate the benefits and risks of medicines [102]. AI can be also used to evaluate the effectiveness of different related strategies and develop and recommend response plans by implementing optimization algorithms in supply chain, which is critical for resiliency and sustainability [103–105].

#### Ethical challenges

AI and its application can bring both potential benefits and ethical challenges for reaching responsible health governance. Particularly the frontier AI fields can impose potential risks to the environment and the future of humans. Therefore, the rapid growth of AI applications strongly needs greater urgency to develop ethical guidelines for considering the societal and ethical impacts of AI in the real world [106]. The Ethical Guidelines for trustworthy AI propose requirements including technical safety, data governance, transparency, and accountability [107]. Moreover, social networks, limited privacy, and security challenges have led to the raising of concerns about the ethical dimensions of AI applications. Therefore, redefining the boundaries of the research should be followed by researchers [108].

According to the definition of ethical responsibility, patients have the right to be informed and any permission should be specific per purpose, be freely given, and unambiguous [109]. Besides, large data sets pose many ethical questions regarding governance [110]. Hence, the principles and guidelines can offer beneficial insights and specific measures regarding ethics must be carefully considered in AI applications [106]. It is crucial to recognize that AI alone cannot guarantee that stakeholders place their full trust in the advances that has to offer. Rather, it can help build and sustain trust by reassuring participants of the security and privacy of their sensitive data [49].

## Conclusions

This scoping review illustrated the use of AI applications across the health system's governance. AI, as a powerful tool, can open a new path at the international and national levels to achieve health goals and indicators that have been targeted for many years. AI is expected to be seen as a tool with new applications and capabilities that can change each component of governance in the health ecosystem, which can eventually facilitate achieving SDG’s health goals and other health-related goals. Nevertheless, more research is needed to determine the precise prerequisites and infrastructure to enforce appropriate AI policy within the health systems.

In conclusion, our research crystalized that there is still a gap in improving the health system's governance regarding AI. To achieve this goal, researchers can develop tools to facilitate automating decision-making processes and improve the quality of outcomes, especially in dynamic environments. Accordingly, AI tools can be used to collect and combine multiple sources of data from various databases to support decision-making. Also, Technical solutions and effective governance goals were identified as the main facilitators of AI policy in the governance of health systems. Besides, in order to achieve better control of the growth and impacts of AI, it is recommended that implementing laws and regulations by governments and policymakers be knowledge-based. This continuing learning approach can be used to address governance problems therefore, as future works, researchers can develop and enhance these mechanisms. Eventually, based on the findings of this research in line with previous studies, we propose minimizing the risks associated with the sharing of sensitive data and increasing transparency. Hence, respect for ethical values as well as understanding the importance of technological progress has to be considered in developing and using AI in health governance.

## Data Availability

All data generated or analyzed during this study are included in this published. article and its supplementary information files.

## Abbreviations

AI: Artificial Intelligence
CEA: Cost-Effectiveness Analysis
GIS: Geographic Information Systems
STM: Structural Topic Modeling
HIM: Health Information Management
SDGs: Sustainable Development Goals
HDE: Health Data Entanglement
IViewOps: Intelligent View Of Operations
GDP: Gross Domestic Product
WHO: World Health Organization
EBOC: Evidence-Based Optimal Control
PLM: Product Lifecycle Management
UHC: Universal Health Coverage
RWD: Real-World Data
HRQoL: Health-Related Quality of Life
